# A kind of system of multivariate variational inequalities and the existence theorem of solutions

**DOI:** 10.1186/s13660-017-1486-9

**Published:** 2017-09-07

**Authors:** Yanxia Tang, Jinyu Guan, Yongchun Xu, Yongfu Su

**Affiliations:** 1 Department of Mathematics, Hebei North University, Zhangjiakou, 075000 China; 2 Department of Mathematics, Tianjin Polytechnic University, Tianjin, 300387 China

**Keywords:** variational inequality, system of variational inequalities, reflexive Banach space, dual space, solution

## Abstract

Let *K* be a nonempty closed convex and bounded subset of a reflexive Banach space *X*. Let $A_{1}, A_{2},\ldots,A_{N}$ be *N*-variables monotone demi-continuous mappings from $K^{N}$ into *X*. Then: (1) the system of multivariate variational inequalities

$$\textstyle\begin{cases} \langle A_{1}(x_{1},x_{2},\ldots,x_{N}), y_{1}-x_{1} \rangle\geq0, &\forall y_{1} \in K,\\ \langle A_{2}(x_{1},x_{2},\ldots,x_{N}), y_{2}-x_{2} \rangle\geq0, &\forall y_{2} \in K,\\ \cdots\\ \langle A_{N}(x_{1},x_{2},\ldots,x_{N}), y_{N}-x_{N} \rangle\geq0, &\forall y_{N} \in K,\\ \end{cases} $$

has a solution $(x_{1}^{*},x_{2}^{*},\ldots,x_{N}^{*}) \in K^{N}$; (2) the set of solutions of this system of multivariate variational inequalities is closed convex in $K^{N}$; (3) if $A_{1}, A_{2},\ldots,A_{N}$ are also strictly monotone, this system of multivariate variational inequalities has a unique solution.

## Introduction

Let *X* be a Banach space with the dual space $X^{*}$ and let $\langle \cdot,\cdot\rangle$ denote the duality pairing of *X* and $X^{*}$. Let *K* be a nonempty closed convex subset of *X*, $A: K\rightarrow X^{*}$ a mapping. The classical variational inequality problem is to find $x \in K$ such that

1.1 $$ \langle Ax, y-x \rangle\geq0, \quad\forall y \in K. $$

The variational inequality problem has been recognized as one of the suitable mathematical models for dealing with many problems arising in different fields, such as optimization theory, game theory, economic equilibrium, mechanics. In the last four decades, since the time of the celebrated Hartman-Stampacchia theorem (see [1, 2]), the existence of a solution of a variational inequality and other related problems has become a basic research topic, which continues to attract attention of researchers in applied mathematics (see *e.g.*, [3–14] and the references therein).

In 1966, Hartman and Stampacchia [2] proved the following result.

### Theorem 1.1

[2]

*Let* *K* *be a nonempty closed convex and bounded subset of* $R^{n}$. *Let* $A: K\rightarrow R^{n}$ *be a continuous mapping*. *Then the variational inequality* (1.1) *has a solution* $x^{*} \in K$.

In 1967, Browder proved the following more general result (see [15]).

### Theorem 1.2

[15]

*Let* *K* *be a nonempty compact convex subset of a locally convex topological vector space* *X*. *Let* $A: K\rightarrow X^{*}$ *be a continuous mapping*. *Then the variational inequality* (1.1) *has a solution* $x^{*} \in K$.

The variational inequality (1.1) is called the Hartman-Stampacchia variational inequality. It is an important classical variational inequality which is also a classical and powerful tool in nonlinear analysis and other mathematical fields.

### Definition 1.3

[15]

Let *X* be a normed space, $A: X\rightarrow X^{*}$ a mapping, $x_{0} \in X$. *A* is said to be demi-continuous at $x_{0}$, if for any given $y\in X$, $A(x_{0}+t_{n}y)$ weak^∗^ converges to $A(x_{0})$ wherever $t_{n}\rightarrow0$, $t_{n}\geq0$.

In 1991, Chang [15] proved the following result in reflexive Banach spaces.

### Theorem 1.4

[15]

*Let* *K* *be a nonempty closed convex and bounded subset of a reflexive Banach space* *X*. *Let* $A: K\rightarrow X^{*}$ *be a monotone demi*-*continuous mapping*. *Then*

1. *the variational inequality* (1.1) *has a solution* $x^{*} \in K$;
2. *the set of solutions of* (1.1) *is closed convex*;
3. *if* *A* *is strictly monotone*, *then* (1.1) *has a unique solution*.

In 2010, Plubtieng and Sombut [16] proved the following result.

### Theorem 1.5

*Let* *X* *be a reflexive Banach space*, *let* *K* *be a compact convex subset of* *X*, *and let* $A,B : K\rightarrow X^{*}$ *be two continuous mappings*. *Then the system of variational inequalities*

1.2 $$ \textstyle\begin{cases} \langle A(x), z-y \rangle\geq0,& \forall z \in K,\\ \langle B(y), z-x \rangle\geq0,& \forall z \in K, \end{cases} $$

*has a solution* $(x,y) \in K\times K$ *and the set of solutions of* (1.2) *is closed*.

Multivariate calculus is a more general mathematical branch which paly a more important role in mathematical and applied fields. In recently, multivariate fixed point theorems and the system of *N*-variables nonlinear operators have been studied by some authors. Many interesting results and the applications have also been given. In 2016, Su *et al.* [17] presented the concept of multivariate fixed point and proved a multivariate fixed point theorem for *N*-variables contraction mappings which further generalizes Banach contraction mapping principle. In 2016, Luo *et al.* [18] presented the concept of multivariate best proximity point and proved the multivariate best proximity point theorems in metric spaces for *N*-variables contraction mappings. In 2017, Xu *et al.* [19] presented the concept of multivariate contraction mapping in a locally convex topological vector spaces and proved the multivariate contraction mapping principle in such spaces. In 2017, Guan *et al.* [20] studied a kind of system of *N*-variables pseudocontractive operator equations and proved the existence theorem of solutions.

The purpose of this paper is to study a kind of system of multivariate variational inequalities and to prove the existence theorem of solutions. The results of this paper improve and extend the results of [15, 16] in reflexive Banach spaces. In order to get the expected results, an ingenious mathematical method is used in this paper.

## Preliminaries

Let us introduce some conclusions which will be useful for our main results.

### Lemma 2.1

[20]

*Let* *X* *be a Banach space with the norm* $\|\cdot\|$. *We consider on the Cartesian product space* $X^{N}=X\times X\times \cdots\times X$ *the following functional*:

$$\|x \|_{*}= \sqrt{\sum_{i=1}^{N}\| x_{i}\|^{2} }, \quad\forall x=(x_{1},x_{2}, \ldots,x_{N}) \in X^{N}. $$

*Then* $(X^{N}, \|\cdot\|_{*})$ *is a Banach space*.

### Lemma 2.2

[20]

$(X^{N},\|\cdot\|_{*})^{*}=((X,\|\cdot\|)^{*})^{N}$.

### Lemma 2.3

[20]

*Let* *X* *be a reflexive Banach space with the norm* $\|\cdot\|$, $X^{N}=X\times X\times \cdots\times X$ *be Cartesian product space of* *X*. *Let*

$$\|x \|_{*}= \sqrt{\sum_{i=1}^{N}\| x_{i}\|^{2} }, \quad\forall x=(x_{1},x_{2}, \ldots,x_{N}) \in X^{N}. $$

*Then* $(X^{N}, \|\cdot\|_{*})$ *is a reflexive Banach space*.

## Main results

Let *K* be a nonempty subset of a normed space *X*, $A_{i}: K^{N}\rightarrow X^{*}$ a *N*-variables mapping for all $i=1,2,\ldots,N$. We consider the following system of multivariate variational inequalities:

3.1 $$ \textstyle\begin{cases} \langle A_{1}(x_{1},x_{2},\ldots,x_{N}), y_{1}-x_{1} \rangle\geq0, &\forall y_{1} \in K,\\ \langle A_{2}(x_{1},x_{2},\ldots,x_{N}), y_{2}-x_{2} \rangle\geq0, &\forall y_{2} \in K,\\ \cdots\\ \langle A_{N}(x_{1},x_{2},\ldots,x_{N}), y_{N}-x_{N} \rangle\geq0, &\forall y_{N} \in K. \end{cases} $$

### Definition 3.1

Let *K* be a nonempty subset of a normed space *X*. A *N*-variables mapping $A: K^{N}\rightarrow X^{*}$ is said to be monotone, if

$$\bigl\langle A(x_{1},x_{2}, \ldots,x_{N})-A(y_{1},y_{2}, \ldots ,y_{N}), x_{i}-y_{i}\bigr\rangle \geq0, \quad\forall i=1,2,\ldots,N, $$

for all $(x_{1},x_{2}, \ldots,x_{N}), (y_{1},y_{2}, \ldots ,y_{N})\in K^{N}$. A *N*-variables monotone mapping is said to be strictly monotone, if

$$\bigl\langle A(x_{1},x_{2}, \ldots,x_{N})-A(y_{1},y_{2}, \ldots ,y_{N}), x_{i}-y_{i}\bigr\rangle = 0, \quad\forall i=1,2,\ldots,N, $$

implies $(x_{1},x_{2}, \ldots,x_{N})=(y_{1},y_{2}, \ldots,y_{N})$.

The following is the main result of this paper.

### Theorem 3.2

*Let* *K* *be a nonempty closed convex and bounded subset of a reflexive Banach space* *X*. *Let* $A_{i}: K^{N}\rightarrow X^{*}$ *be a* *N*-*variables monotone demi*-*continuous mapping for all* $i=1,2,\ldots,N$. *Then*:

1. *the system of variational inequalities* (3.1) *has a solution* $(x_{1}^{*},x_{2}^{*}, \ldots,x_{N}^{*}) \in K^{N}$;
2. *the set of solutions of* (3.1) *is closed convex in* $K^{N}$;
3. *if* $A_{i}$ *is strictly monotone for all* $i=1,2,\ldots,N$, *then* (3.1) *has a unique solution*.

### Proof

Let $A^{*}:K^{N} \rightarrow(X^{*})^{N}$ be a mapping defined by

$$A^{*}(x_{1},x_{2}, \ldots,x_{N})= \bigl(A_{1}(x), A_{2}(x), \ldots, A_{N}(x) \bigr), $$

for any $x=(x_{1},x_{2}, \ldots,x_{N})\in K^{N} $, where

$$\begin{gathered} A_{1}(x)= A_{1}(x_{1},x_{2}, \ldots,x_{N}), \\ A_{2}(x)=A_{2}(x_{1},x_{2}, \ldots,x_{N}), \\ \cdots \\ A_{N}(x)=A_{N}(x_{1},x_{2}, \ldots,x_{N}). \end{gathered} $$

From Lemma 2.1 and Lemma 2.2, we know $(X^{*})^{N}=(X^{N})^{*}$ and hence $A^{*}$ is a mapping from $K^{N}$ into $(X^{N}, \|\cdot\|_{*})^{*}$.

Next, we prove that $A^{*}$ is a monotone mapping from $K^{N}$ into $(X^{N}, \|\cdot\|_{*})^{*}$. Since $A_{i}$ is a monotone mapping from *K* into $(X, \|\cdot\|)^{*}$ for all $i=1,2,\ldots,N$,

$$\begin{gathered} \bigl\langle A^{*}x-A^{*}y, x-y \bigr\rangle \\ \quad=\bigl\langle \bigl(A_{1}(x)-A_{1}(y), A_{2}(x)-A_{2}(y), \ldots, A_{N}(x)-A_{N}(y)\bigr), x-y \bigr\rangle \\ \quad=\sum_{i=1}^{N} \bigl\langle A_{i}(x)-A_{i}(y), x_{i}-y_{i}\bigr\rangle \geq0, \end{gathered} $$

for any $x=(x_{1},x_{2}, \ldots,x_{N}), y=(y_{1},y_{2}, \ldots ,y_{N})\in K^{N} $.

We also need to prove $A^{*}$ is demi-continuous on $K^{N}$. For any given $x_{0} \in K^{N}$ and any given $y=(y_{1},y_{2}, \ldots,y_{N}) \in K^{N}$ such that $x_{0}+t_{n}y\in K^{N}$, we have

$$\begin{aligned} A^{*}(x_{0}+t_{n}y) (x)&=\bigl\langle \bigl(A_{1}(x_{0}+t_{n}y), A_{2}(x_{0}+t_{n}y),\ldots ,A_{N}(x_{0}+t_{n}y) \bigr), x \bigr\rangle \\ &= \sum_{i=1}^{N} \bigl\langle A_{i}(x_{0}+t_{n}y), x_{i}\bigr\rangle \\ &\rightarrow \sum_{i=1}^{N} \bigl\langle A_{i}(x_{0}), x_{i}\bigr\rangle \quad(t_{n} \rightarrow0) \\ &= A^{*}(x_{0}) (x), \quad\forall x=(x_{1},x_{2}, \ldots,x_{N}) \in K^{N}. \end{aligned} $$

Then $A^{*}$ is demi-continuous on $K^{N}$.

It is easy to see that $K^{N}$ is a nonempty closed convex and bounded subset of Banach space $(X^{N}, \|\cdot\|_{*})$. By using Theorem 1.4, we know that the following variational inequality:

3.2 $$ \bigl\langle A^{*}x, y-x\bigr\rangle \geq0, \quad\forall y=(y_{1},y_{2}, \ldots ,y_{N}) \in K^{N}, $$

has a solution $x^{*}=(x_{1}^{*},x_{2}^{*}, \ldots,x_{N}^{*})\in K^{N}$. That is,

3.3 $$ \bigl\langle A^{*}x^{*}, y-x^{*}\bigr\rangle \geq0, \quad\forall y=(y_{1},y_{2}, \ldots,y_{N}) \in K^{N}. $$

We rewrite (3.3) as follows:

3.4 $$ \sum_{i=1}^{N}\bigl\langle A_{i} \bigl(x_{1}^{*},x_{2}^{*}, \ldots,x_{N}^{*} \bigr), y_{i}-x_{i}^{*}\bigr\rangle \geq0,\quad \forall y=(y_{1},y_{2}, \ldots ,y_{N}) \in K^{N}. $$

For any $y \in K$, let $y_{i}=(y,x_{2}^{*}, \ldots,x_{N}^{*}) \in K^{N}$ in (3.4), we get

3.5 $$ \bigl\langle A_{1}\bigl(x_{1}^{*},x_{2}^{*}, \ldots,x_{N}^{*}\bigr), y-x_{1}^{*}\bigr\rangle \geq0, \quad\forall y \in K. $$

For any $y \in K$, let $y_{i}=(x_{1}^{*}, \ldots,x_{j-1}^{*}, y, x_{j+1}^{*}, \ldots,x_{N}^{*}) \in K^{N}$ in (3.4), we get

3.6 $$ \bigl\langle A_{j}\bigl(x_{1}^{*},x_{2}^{*}, \ldots,x_{N}^{*}\bigr), y-x_{j}^{*}\bigr\rangle \geq0,\quad \forall y \in K, $$

for all $j=2,3,\ldots, N-1$. For any $y \in K$, let $y_{i}=(x_{1}^{*}, x_{2}^{*},\ldots,x_{N-1}^{*}, y) \in K^{N}$ in (3.4), we get

3.7 $$ \bigl\langle A_{N}\bigl(x_{1}^{*},x_{2}^{*}, \ldots,x_{N}^{*}\bigr), y-x_{N}^{*}\bigr\rangle \geq0,\quad \forall y \in K. $$

From (3.5)-(3.7), we know that $x^{*}=(x_{1}^{*},x_{2}^{*}, \ldots ,x_{N}^{*})$ is a solution of (3.1). This completes the proof of conclusion (1).

On the other hand, let $x=(x_{1},x_{2}, \ldots,x_{N})$ be an arbitrary solution of (3.1). We have

$$\textstyle\begin{cases} \langle A_{1}(x_{1},x_{2},\ldots,x_{N}), y_{1}-x_{1} \rangle\geq 0, &\forall y_{1} \in K,\\ \langle A_{2}(x_{1},x_{2},\ldots,x_{N}), y_{2}-x_{2} \rangle\geq 0, &\forall y_{2} \in K,\\ \cdots\\ \langle A_{N}(x_{1},x_{2},\ldots,x_{N}), y_{N}-x_{N} \rangle\geq 0, &\forall y_{N} \in K, \end{cases} $$

which implies

$$\begin{aligned} \bigl\langle A^{*}x, y-x \bigr\rangle &= \bigl\langle (A_{1}x, A_{2}x,\ldots,A_{N}x), y-x \bigr\rangle \\ &= \sum_{i=1}^{N}\bigl\langle A_{i}(x_{1},x_{2},\ldots,x_{N}), y_{i}-x_{i} \bigr\rangle \geq0, \quad\forall y \in K^{N}. \end{aligned} $$

Then $x=(x_{1},x_{2}, \ldots,x_{N})$ is a solution of the variational inequality (3.2) in reflexive Banach space $(X^{N}, \|\cdot\| _{*})$. From the above, we claim that the system of multivariate variational inequalities (3.1) is equivalent to the variational inequality (3.2). By using Theorem 1.4, we know that the set of solutions of the variational inequality (3.2) is closed convex. This completes the proof of conclusion (2).

Finally, if *A* is strictly monotone, then

$$\bigl\langle A^{*}x-A^{*}y, x-y\bigr\rangle =0, $$

implies $x=y$. Hence $A^{*}$ is also strictly monotone. By using Theorem 1.4, the variational inequality (3.2) has a unique solution and hence the multivariate variational inequalities (3.1) has a unique solution. This completes the proof. □

### Corollary 3.3

*Let* *K* *be a nonempty closed convex and bounded subset of a reflexive Banach space* *X*. *Let* $A: K\rightarrow X^{*}$ *be a* *N*-*variables monotone semi*-*continuous mapping*. *Then*:

1. *the multivariate variational inequalities*
  3.8 $$ \bigl\langle A(x_{1},x_{2}, \ldots,x_{N}), y-x_{i}\bigr\rangle \geq0, \quad\forall y \in K, \forall i=1,2,\ldots,N $$
  *has a solution* $(x_{1}^{*},x_{2}^{*}, \ldots,x_{N}^{*}) \in K^{N}$;
2. *the set of solutions of* (3.8) *is closed convex in* $K^{N}$;
3. *if* *A* *is strictly monotone*, *then* (3.8) *has a unique solution*.

### Proof

Let $A_{i}=A$ for all $i=1,2,\ldots,N$ in Theorem 3.2, we can get the conclusion. □

### Corollary 3.4

*Let* *K* *be a nonempty closed convex and bounded subset of a reflexive Banach space* *X*. *Let* $A: K\rightarrow X^{*}$ *be a* *N*-*variables monotone semi*-*continuous mapping*. *Then*

1. *the multivariate variational inequalities*
  3.9 $$ \Biggl\langle A(x_{1},x_{2}, \ldots,x_{N}), y-\frac{1}{N}\sum_{i=1}^{N}x_{i} \Biggr\rangle \geq0,\quad \forall y \in K, $$
  *has a solution* $(x_{1}^{*},x_{2}^{*}, \ldots,x_{N}^{*}) \in K^{N}$;
2. *the set of solutions of* (3.9) *is closed convex in* $K^{N}$;
3. *if* *A* *is strictly monotone*, *then* (3.9) *has a unique solution*.

### Proof

From (3.8), we have

$$\frac{1}{N}\sum_{i=1}^{N}\bigl\langle A(x_{1},x_{2}, \ldots,x_{N}), y-x_{i}\bigr\rangle \geq0, \quad\forall y \in K. $$

That is,

$$\Biggl\langle A(x_{1},x_{2}, \ldots,x_{N}), y-\frac{1}{N}\sum_{i=1}^{N}x_{i} \Biggr\rangle \geq0,\quad \forall y \in K. $$

This completes the proof. □

Next, we prove an existence theorem of solutions for the system of variational inequalities (1.2) in normed spaces.

### Theorem 3.5

*Let* *X* *be a normed space*, *let* *K* *be a compact convex subset of* *X*, *and let* $A,B : K\rightarrow X^{*}$ *be two continuous mappings*. *Then the system of variational inequalities* (1.2) *has a solution* $(x,y) \in K\times K$ *and the set of solutions of* (1.2) *is closed*.

### Proof

Let $A(x,y)=A(y)$, $B(x,y)=B(x)$ for all $(x,y) \in K\times K$, then the system of variational inequalities (1.2) is equivalent to

3.10 $$ \textstyle\begin{cases} \langle A(y, x), z-y \rangle\geq0,& \forall z \in K,\\ \langle B(y, x), z-x \rangle\geq0,& \forall z \in K. \end{cases} $$

Let $C^{*}: K \times K \rightarrow X^{*} \times X^{*}=(X\times X)^{*}$ be defined by

$$C^{*}(x,y)=\bigl(A(x,y),B(x,y)\bigr) $$

for all $(x,y) \in K\times K$. It is easy to see that $C^{*}$ is a continuous mapping from the nonempty compact convex subset $K\times K$ into the dual space $(X \times X)^{*}$ of normed space $X \times X$. By using Theorem 1.2, there exists an element $(x^{*}, y^{*}) \in K \times K$ such that

$$\bigl\langle C^{*}\bigl(x^{*},y^{*}\bigr), (z_{1},z_{2})- \bigl(x^{*},y^{*}\bigr)\bigr\rangle _{*} \geq0, \quad\forall (z_{1},z_{2}) \in K\times K, $$

where $\langle\cdot,\cdot\rangle_{*}$ denotes the duality pairing of $X\times X$ and $X^{*} \times X^{*}=(X\times X)^{*}$. This implies

$$\bigl\langle \bigl(A\bigl(x^{*},y^{*}\bigr), B\bigl(x^{*},y^{*}\bigr)\bigr), \bigl(z_{1}-x^{*},z_{2}-y^{*}\bigr)\bigr\rangle _{*} \geq0, \quad\forall (z_{1},z_{2}) \in K\times K. $$

Hence

$$\bigl\langle \bigl(A\bigl(y^{*}\bigr), B\bigl(x^{*}\bigr)\bigr), \bigl(z_{1}-x^{*},z_{2}-y^{*}\bigr)\bigr\rangle _{*} \geq0, \quad\forall (z_{1},z_{2}) \in K\times K. $$

From the definition of $\langle\cdot, \cdot\rangle_{*}$, we have

3.11 $$ \bigl\langle A\bigl(y^{*}\bigr), z_{1}-x^{*}\bigr\rangle +\bigl\langle B \bigl(x^{*}\bigr), z_{2}-y^{*}\bigr\rangle \geq0, \quad\forall (z_{1},z_{2}) \in K\times K. $$

Let $z_{2}=y^{*}$ and $z_{1}=x^{*}$ in (3.11), respectively, we get

$$\textstyle\begin{cases} \langle A(y^{*}), z_{1}-x^{*} \rangle\geq0,& \forall z_{1} \in K,\\ \langle B(x^{*}), z_{2}-y^{*} \rangle\geq0, &\forall z_{2} \in K. \end{cases} $$

Then $(x^{*},y^{*}) \in K \times K$ is a solution of the system of variational inequalities (1.2). Since *A*, *B* are continuous, so the set of solutions of (1.2) is closed. This completes the proof. □

It is obvious that Theorem 1.5 is a special form of Theorem 3.5 in reflexive Banach spaces.

### Corollary 3.6

Theorem 1.5

*Let* *X* *be a reflexive Banach space*, *let* *K* *be a compact convex subset of* *X*, *and let* $A,B : K\rightarrow X^{*}$ *be two continuous mappings*. *Then the system of variational inequalities*

$$\textstyle\begin{cases} \langle A(x), z-y \rangle\geq0, &\forall z \in K,\\ \langle B(y), z-x \rangle\geq0, &\forall z \in K, \end{cases} $$

*has a solution* $(x,y) \in K\times K$ *and the set of solutions of* (1.2) *is closed*.

We give an example to show the mathematical and physical significance of the main results of this paper.

### Example 3.7

Let $R=(-\infty,+\infty)$, $K=[a,b]$. Let $f(x_{1},x_{2},\ldots,x_{N})$ be a continuous real *N*-variables function with $f \in C^{(1)}(K^{N}, R)$. Then there exists an element $x_{0}= (x_{0,1}, x_{0,2}, \ldots,x_{0,N}) \in K^{N}$ such that

$$f(x_{0,1}, x_{0,2}, \ldots,x_{0,N})=\min _{(x_{1},x_{2},\ldots,x_{N}) \in K^{N}}f(x_{1},x_{2},\ldots,x_{N}). $$

This element $x_{0}$ must be a solution of the following system of multivariate variational inequalities:

3.12 $$ \textstyle\begin{cases} \langle\frac{\partial f}{\partial x_{1}}(x_{1},x_{2},\ldots ,x_{N}), y_{1}-x_{1} \rangle\geq0, &\forall y_{1} \in K,\\ \langle\frac{\partial f}{\partial x_{2}}(x_{1},x_{2},\ldots ,x_{N}), y_{2}-x_{2} \rangle\geq0, &\forall y_{2} \in K,\\ \cdots\\ \langle\frac{\partial f}{\partial x_{N}}(x_{1},x_{2},\ldots ,x_{N}), y_{N}-x_{N} \rangle\geq0, &\forall y_{N} \in K. \end{cases} $$

In fact, we have

$$\frac{\partial f}{\partial x_{i}}(x_{0,1},x_{0,2},\ldots,x_{0,N}) \textstyle\begin{cases} = 0, &x_{0,i} \in(a,b),\\ \geq0,& x_{0,i}=a,\\ \leq0,& x_{0,i}=b, \end{cases} $$

for all $i=1,2,\ldots,N$. Hence $x_{0}$ must satisfy (3.12). In addition, the system of multivariate variational inequalities (3.12) is equivalent to

3.13 $$ \bigl\langle \operatorname{grad} f(x), y-x \bigr\rangle \geq0, \quad\forall y \in K^{N}, $$

where

$$\operatorname{grad} f(x)=\biggl(\frac{\partial f}{\partial x_{1}}, \frac{\partial f}{\partial x_{2}},\ldots, \frac{\partial f}{\partial x_{N}}\biggr). $$

This example is actually a practical background of Theorem 3.2, where $A_{i}=\frac{\partial f}{\partial x_{i}}$ for all $i=1,2,\ldots,N$ and $\operatorname{grad} f(x)=A^{*}$.

## Conclusion

In this article, we use an ingenious mathematical method to prove the existence theorem of solutions for a kind of system of multivariate variational inequalities:

$$\textstyle\begin{cases} \langle A_{1}(x_{1},x_{2},\ldots,x_{N}), y_{1}-x_{1} \rangle\geq0, &\forall y_{1} \in K,\\ \langle A_{2}(x_{1},x_{2},\ldots,x_{N}), y_{2}-x_{2} \rangle\geq0, &\forall y_{2} \in K,\\ \cdots\\ \langle A_{N}(x_{1},x_{2},\ldots,x_{N}), y_{N}-x_{N} \rangle\geq0, &\forall y_{N} \in K. \end{cases} $$

Here *K* is a nonempty closed convex and bounded subset of a reflexive Banach space *X* and $A_{1}, A_{2},\ldots,A_{N}$ are *N*-variables monotone demi-continuous mappings from $K^{N}$ into $X^{*}$. This system of multivariate variational inequalities has a solution. The set of solutions of this system of multivariate variational inequalities is closed convex in $K^{N}$. If $A_{1}, A_{2},\ldots,A_{N}$ are also strictly monotone, this system of multivariate variational inequalities has a unique solution.
